# Endometrioid adenofibroma of ovary: A case report and review of literature

**DOI:** 10.1097/MD.0000000000032965

**Published:** 2023-02-22

**Authors:** Hai-Chao Tong, Ying-Chun Li, Le-Yao Li, Hong-Tao Xu, Shuang Ma, Wan-Lin Zhang, Tyler Wildes, Lian-He Yang, Endi Wang

**Affiliations:** a Department of Pathology, the First Hospital and College of Basic Medical Sciences, China Medical University, Shenyang, Liaoning, China; b Department of Neurology, Sheng Jing Hospital of China Medical University, Shenyang, Liaoning, China; c Department of Pathology, Hebei Petro China Central Hospital, Langfang, Hebei, China; d Department of Pathology, Duke University Medical Center, Durham, North Carolina, USA.

**Keywords:** cystadenofibroma, endometrioid adenofibroma, endometriosis, ovarian neoplasms, serous adenofibroma

## Abstract

**Rationale:**

Endometrioid adenofibroma is a benign epithelial neoplasm of the ovary, most of which are often unilateral. The symptoms of endometrioid adenofibroma are often nonspecific and misleading. Therefore, a full understanding of the characteristics, diagnosis, and treatment methods of this disease is of great importance. In this study, we report a 34-year-old woman who was found with an unidentified mass on the right ovary during the physical examination 3 years ago with nosymptoms or signs.

**Patient concerns:**

A 34-year-old Chinese female was found with an unidentified 6 cm mass on the right ovary for 3 years that presented with no symptoms or signs.

**Diagnosis:**

Pelvic ultrasound revealed a 6 cm cystic solid mixed mass on the right ovary. Through histological and immunohistochemical examinations, the tumor mass was finally diagnosed as endometrioid adenofibroma of ovary.

**Interventions:**

To confirm the diagnosis, the ovarian tumor was laparoscopically resected.

**Outcomes:**

The patient returned to hospital after 3 months with no recurrence or postoperative complications.

**Lessons:**

Endometrioid adenofibroma is a benign epithelial neoplasm of the ovary. Complete surgical resection is required and rare cases can recur. Postsurgical pathologic and immunohistochemical testing can confirm a diagnosis of endometrioid adenofibroma. It is important to understand of the key points of differential diagnosis of the disease due to the different prognosis and clinical treatment.

## 1. Introduction

Ovarian tumors are common tumors of the female reproductive system. According to the 2020 World Health Organization classification of tumors of female reproductive organs, ovarian endometrioid tumors can be classified into endometrioid cystadenoma and adenofibroma, endometrioid borderline tumor (EBT), endometrioid adenocarcinoma, and seromucinous carcinoma according to histopathological characteristics. Among these, endometrioid cystadenoma and adenofibroma are benign epithelial tumors with endometrioid differentiation and are rarely seen in clinic.^[1]^ Endometrioid cystadenoma is a cystic lesion lined by benign endometrioid epithelium, and endometrioid adenofibroma is supported when accompanied by a dense fibroid component. But when there is a large cystic space within the gland, endometrioid cystadenofibroma is an appropriate name. Clinical patients with this disease may present with pelvic mass-related symptoms, or discover the tumor by chance. Here we report a 34-year-old female patient with benign endometrioid adenofibroma of the ovary and discuss the clinical characteristics, pathological diagnosis and treatment of this tumor.

## 2. Case presentation

A 34-year-old female patient went to a local hospital for treatment with “ovarian tumors found on physical examination for three years,” denying any abnormal symptoms with normal menstruation, no vaginal discharge and no contact bleeding. In addition, the patient also denied a history of hypertension, coronary heart disease, diabetes, or acute and chronic infectious diseases. The physical examination showed the patient’s general condition was good and her vital signs were stable. Serum tumor makers were tested and demonstrated carbohydrate antigen 125: 117.9 μL/mL (0.00~35.00 μL/mL) and alpha fetoprotein: 11.5 ng/mL (0.00~4.30 ng/mL). Pelvic ultrasound and computed tomography showed a 6 cm cystic solid mixed mass in the right ovary with multiple uterine microfibroids, adenomyosis, and a small amount of fluid in the pelvic cavity. The patient was TCT negative (−) and HPV negative (−) and underwent a laparoscopic right ovarian tumor resection.

**Figure 1.**
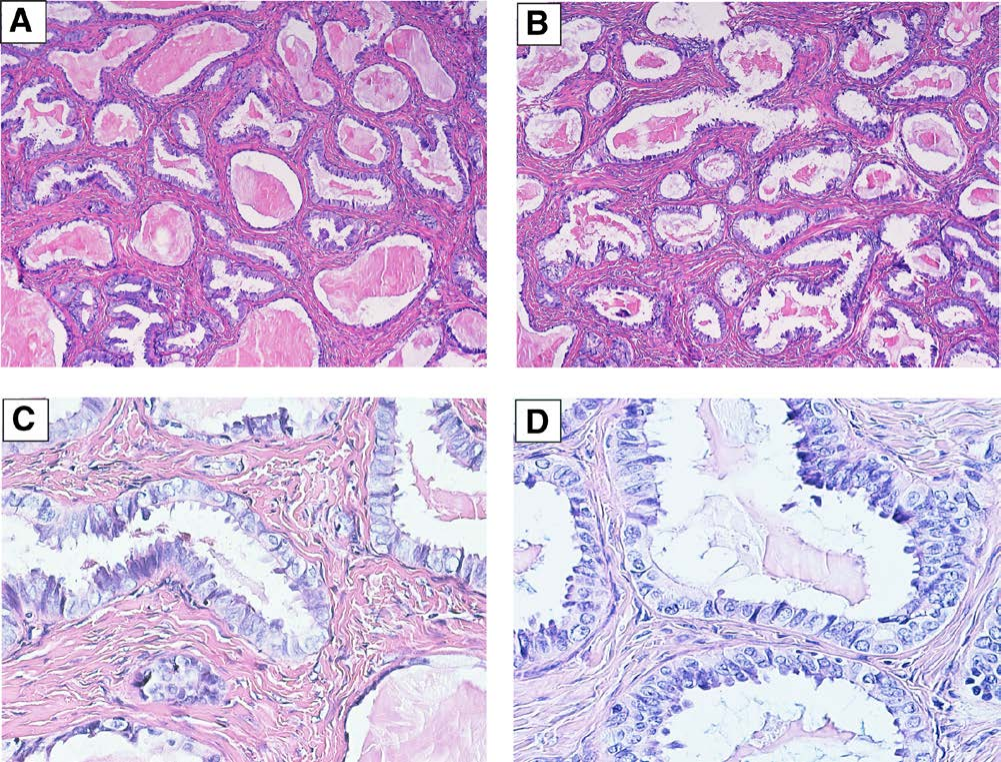
(A and B) Endometrioid glands of different sizes are scattered in dense fibrous stroma. There are red stained secretions in the gland cavity (100×). (C and D) High power microscopy shows that the glandular epithelial cells are tall columnar with cilia, the nucleus is oval with small nucleoli, and the cytoplasm is biphilic or slightly basophilic (400×).

**Figure 2.**
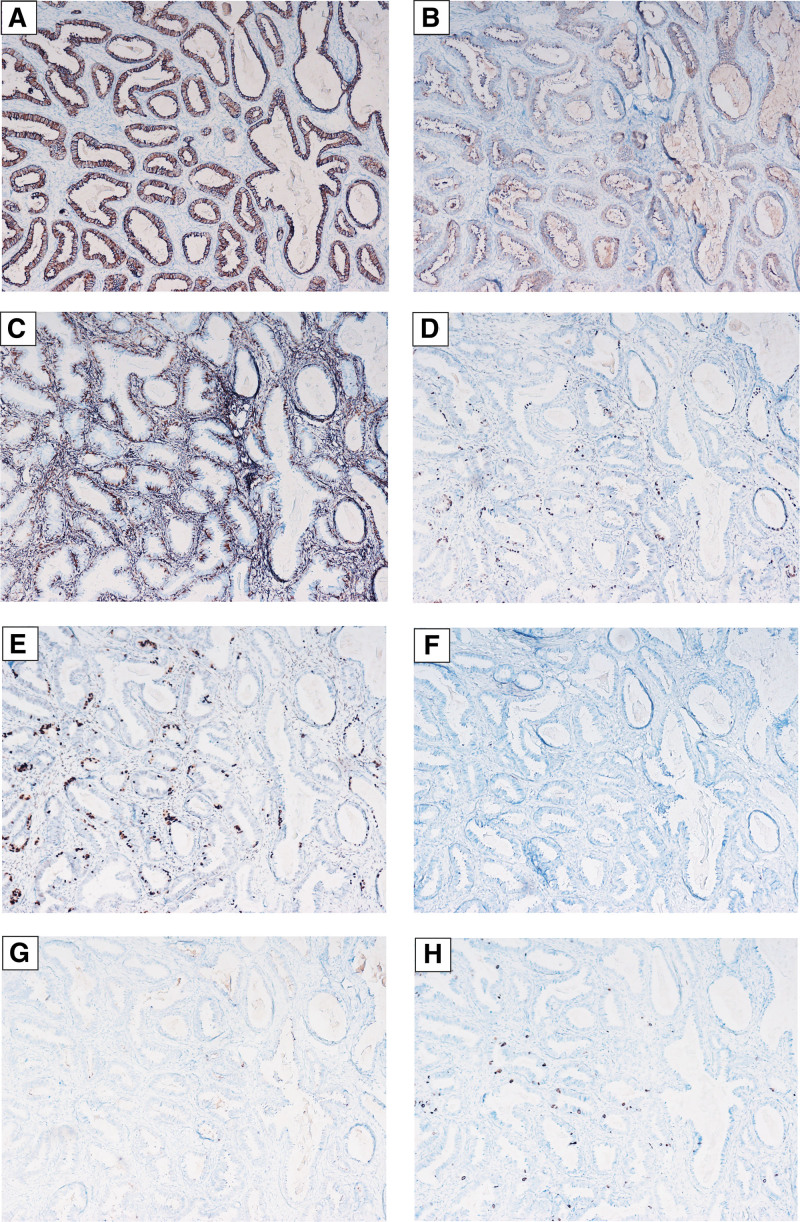
(A) CK stain showed positive expression (100×). CK = cytokeratin. (B) EMA showed positive expression (100×). EMA = epithelial membrane antigen. (C) Vimentin showed positive expression (100×). (D and E) partially positive expression for ER and PR (100×). ER = estrogen receptor; PR= progesterone receptor. (F and G) CD10 and WT-1 showed negative expression (100×). (H) Ki-67 proliferation index is about 2% (100×).

The gross examination showed that the tumor is about 6 cm in diameter, the surface of the tumor was smooth, and its cut surface was grayish red to grayish white. Some areas had cystic change which showed honeycomb appearance with yellow liquid inside. The postsurgical histologic examination showed endometrioid glands scattered in dense fibrous stroma with a lack of endometrial-type stromaunder the epithelium (Fig. 1A and B). Under high magnification, the epithelial cells are tall columnar and arranged in a single layer or a pseudo-stratified layer, and cilia are visible on the luminal surface (Fig. 1A and B). The nuclei are oval with inconspicuous nucleoli (Fig. 1C and D). Immunohistochemical tests of intimal epithelium demonstrated diffuse positive expression of CK, EMA and Vimentin, partially positive expression for ER and PR, and negative expression for CD10 and WT-1 (Fig. 2). The Ki-67 proliferation index was about 2%. According to the clinical and pathological presentation, the tumor was diagnosed as endometrioid adenofibroma of ovary. The patient recovered well and was discharged after surgery without any complications. The follow-up time was 2 years, and there was no recurrence or metastasis of the tumor.

## 3. Discussion

Adenofibroma is a rare benign Mullerian mixed tumor, which was first proposed by the pathologist Ober in 1959.^[2]^ Its histological types are divided into endometrioid, serous, mucinous, clear cell and mixed types. So far, about 40 cases of adenofibroma have been reported in the literature, most of which were reported as individual cases. Endometrioid adenofibromas are rare benign tumors, accounting for 1% of the epithelial neoplasm of the ovary, and 83% are unilateral. The median age of this disease is 57 years old, encompassing largely postmenopausal women.^[3,4]^ This case is reported in a 34-year-old woman of reproductive age. It also occurred in a 17-year-old female who was reported to have a large solid and cystic ovarian mass and the histology revealed a proliferating endometrioid cystadenofibroma.^[5,6]^ According to statistics, about 90% of adenofibromas occur in the endometrium and cervical mucosa, and 10% originate from other parts of the uterus. It is rare to occur in the ovary, and ovarian adenofibromas are even rarer.^[7,8]^

Since the clinical presentation of endometrioid adenofibroma is nonspecific, the diagnosis of this disease is particularly challenging, especially to inexperienced clinicians. The most common presenting symptom is abdominal pain. Abnormal vaginal bleeding has been reported in few cases of cystadenofibromas.^[6]^ Acute pain may also occur when there is a relatively large cyst torsion or blood collection into the potential or adjacent endometriotic cyst.^[9]^

At gross examination, endometrioid adenofibroma are solid or cystic, with an average diameter of about 10 cm. The outer surface is smooth, and the section is densely fibrous, among which are scattered lumen of different sizes, containing transparent or light yellow liquid in them. The cyst wall is smooth and there are a few nodular protrusions of different size.^[3]^

Microscopically, endometrioid adenofibroma is mainly composed of fibrous interstitium, among which are scattered endometrioid glands. The gland components are made up of tubular and cystic glands of different sizes lined with benign hyperplastic epithelium, and the glandular epithelium is a single layer of cuboidal or low columnar cells. Dystrophic calcification is sometimes seen and squamous differentiation in the form of squamous morules can occur. As with other endometrioid tumors of the ovary, these tumors may occur within an endometriosis cyst.^[9,10]^

On ultrasound, adenofibroma or cystadenofibroma has a different solid and cystic appearance, and the solid component can show the posterior sound shadow coming from dense fibrous tissue.^[11]^ In computed tomography scanning, adenofibroma showed solid, cystic or cystic lesions with clear boundaries, and spacelike structures were found in some of the lesions, which were mainly related to the components of the glands and fibrous matrix and the secretory activities of the glands. The liquid-liquid plane is visible when the cystic component is co-deposited.^[12]^ Magnetic resonance imaging (MRI) is considered the preferred modality for imaging of complex ovarian masses. At MRI, the solid component exhibits characteristic low T2 signal intensity, which is lower than that of muscle and corresponds to the fibrous stroma. Scattered cysts with sponge like appearance can also be seen at MRI.^[6,13,14]^

Endometrioid ovarian tumors are very similar to endometrioid tumors of the uterine corpus. They are often related to endometriosis in the same ovary and/or other sites, and in some cases they appear in an endometriosis cyst, indicating its origin in endometriosis. Some case reports indicate that endometriosis is found in endometrioid adenofibromas. In other cases, they can be present together with endometrial tumor, which indicates the same risk factors.^[15,16]^ Ovarian endometriosis can be seen as a origin of the tumor. Tumors caused by endometriosis have been called EANs (***definition), the most common of which are clear cell tumors and endometrioid tumors (most commonly cancerous but occasionally borderline or benign tumors). These are more likely to occur in the ovaries than in extraovarian endometriosis.^[17,18]^

Cytological findings of endometrioid ovarian adenofibroma (fine needle aspiration and tumor tissue imprint) show benign endometrial-like epithelial cells and spindle stromal cells.^[15]^ In contrast to endometriosis, mitoses are rarely seen in the epithelial component, while the absence of endometrial-type stroma and hemosiderin-laden macrophages further distinguish adenofibroma from endometriosis.

In addition, we should distinguish the endometrioid adenofibroma of the ovary from other ovarian tumors. EBT are thought to originate from adenofibroma or endometriosis.^[19]^ In contrast to benign endometrioid adenofibroma, EBT show varying degrees of atypical, crowded, back-to-back, or sieve like glands that resemble atypical hyperplasia in the dense fibrous interstitium, occasionally in a papillary pattern, and may show foci of confluent or infiltrative microinvasion (<5 mm). Squamous morules are often present in EBT with reduced ER and PR expression, which helps distinguish them from endometrioid adenofibromas with solid growth foci.^[20]^

Among ovarian benign adenofibromas, serous adenofibroma is the most common. According to recent studies, serous cystadenoma is the most common ovarian tumor and is easily misinterpreted as endometrioid adenofibroma.^[14,18,21]^ Sometimes it may be difficult to distinguish between serous and endometrioid-type epithelium. The distinction between them is subjective as they both have ciliated epithelium.^[22]^ But some signs can help us, the absence of multiple cysts and elongated papillary tubular glandular structure of serous gonadadous fibromas support endometrioid differentiation.

In contrast to endometrioid adenofibroma, the tumor cells of ovarian endometrioid adenocarcinoma show distinct atypia, easy to see mitotic images, and invasive growth. Additionally, in differentiation from ovarian granulosa cell tumor, we should note that ovarian granulosa cell tumor is usually well demarcated and is a round or lobulated multilocular cystic or solid cystic mass with more bleeding within the mass. Due to the scattered solid and cystic parts of the tumor, it shows a “spongy” appearance on imaging.^[23]^

Surgery is the main treatment of endometrioid adenofibroma, the treatment effect can be achieved by complete removal of the ovaries and fallopian tubes or the tumor, and rare cases can recur.

In conclusion, endometrioid adenofibroma is a rare benign epithelial tumor of the ovary, the exact incidence is not known and the clinical symptoms are usually nonspecific. Meanwhile, due to the different preoperative imaging and pathological findings, misdiagnosis and mistreatment are common. The possibility of adenofibroma should be considered when a heterogeneous, polycystic mass with clear boundaries is found on imaging, but the nature of the tumor is determined by histopathology. Therefore, in clinical diagnosis of endometrioid adenofibroma of ovary, it is important to strengthen its pathological examination, and improve the diagnostic accuracy. Although there is no clinical evidence of malignancy in adenofibromas, it is recommended to remove the adnexa when the lesion is diagnosed to prevent tumor recurrence. Patients should be followed up regularly to monitor the prognosis.

## Author contributions

**Conceptualization:** Hai-Chao Tong, Wan-Lin Zhang.

**Data curation:** Ying-Chun Li.

**Formal analysis:** Hai-Chao Tong, Shuang Ma.

**Funding acquisition:** Lian-He Yang.

**Investigation:** Ying-Chun Li, Shuang Ma.

**Methodology:** Hong-Tao Xu.

**Project administration:** Lian-He Yang.

**Resources:** Le-Yao Li.

**Software:** Le-Yao Li.

**Supervision:** Endi Wang.

**Validation:** Hong-Tao Xu.

**Visualization:** Wan-Lin Zhang.

**Writing – original draft:** Hai-Chao Tong.

**Writing – review & editing:** Tyler Wildes, Lian-He Yang.
